# Barriers and Priorities for Immunotherapy Implementation in Advanced Gastric Cancer: Findings from a Two-Phase Qualitative Study

**DOI:** 10.1007/s12029-026-01557-0

**Published:** 2026-09-03

**Authors:** Angela Ragonese, Mario Airoldi, Maria Costanza Aquilano, Graziana Arborea, Katia Bencardino, Federica Castri, Ferdinando De Vita, Claudio Lotesoriere, Iacopo Panarese, Paolo Pochettino, Maria Antonietta Satolli, Antonia Strippoli, Annamaria Valentini, Paolo Sciattella, MAGIC Working Group, Giulia Caira, Loretta Cervi, Alessandro Cioce, Francesco Leone, Fausto Rosa, Angela Dalia Ricci, Maria Savino, Maria Scatolini, Elisa Sperti

**Affiliations:** 1 Economic Evaluation and HTA (EEHTA CEIS) –University of Rome “Tor Vergata”, Rome, Italy; 2 Medical Oncology 2U, Azienda Ospedaliero-Universitaria Città Della Salute E Della Scienza Di Torino, Turin, Italy; 3 Surgical Pathology Unit, Niguarda Cancer Centre, ASST Grande Ospedale Metropolitano Niguarda, Milan, Italy; 4 Department of Pathology, National Institute of Gastroenterology, IRCCS S. de Bellis Research Hospital, Castellana Grotte, Bari, Italy; 5 Department of Haematology, Oncology and Molecular Medicine, Niguarda Cancer Centre, Grande Ospedale Metropolitano Niguarda, Milan, Italy; 6 Division of Anatomic Pathology and Histology, Fondazione Policlinico Universitario A. Gemelli-IRCCS, Università Cattolica del Sacro Cuore, Rome, Italy; 7 Division of Medical Oncology, Department of Precision Medicine, School of Medicine, University of Study of Campania Luigi Vanvitelli, Naples, Italy; 8 Medical Oncology Unit, National Institute of Gastroenterology, IRCCS S. de Bellis Research Hospital, Castellana Grotte, Bari, Italy; 9 Pathology Unit, University of Campania Luigi Vanvitelli, Naples, Italy; 10 Medical Oncology 1U, Azienda Ospedaliero-Universitaria Città Della Salute E Della Scienza Di Torino, Turin, Italy; 11 Medical Oncology, Comprehensive Cancer Centre, Fondazione Policlinico Universitario Agostino Gemelli IRCCS, Rome, Italy

**Keywords:** Advanced Gastric Cancer, Immunotherapy, Biomarker Testing, Diagnostic Pathways, Multidisciplinary Care, Qualitative Research

## Abstract

**Background:**

Immunotherapy has changed the therapeutic approach to advanced gastric cancer (AGC) and has shown survival benefits compared with standard chemotherapy. However, in clinical practice, its use remains heterogeneous. Differences in diagnostic pathways, organisational aspects, and regional settings may lead to delays in treatment access. This study aimed to analyse these issues and to describe possible shared strategies to improve the use of immunotherapy in AGC in Italy.

**Materials and Methods:**

The analysis was performed in two steps. First, a focus group comprising 13 oncologists and pathologists from 5 national reference centres was organised to collect information on the use of immunotherapy biomarker tests and the main barriers encountered in daily practice. Based on the topics emerging from this discussion, a second step was conducted using a consensus questionnaire. The questionnaire was sent to a multidisciplinary group composed of 19 oncologists, pathologists, surgeons, pharmacists, and endoscopists.

**Results:**

Several common problems were reported during the focus group discussion. These included delays in biomarker testing, differences in pre-analytical procedures among centres, the absence of shared quality indicators, and difficulties in tracking tissue samples, especially when samples were sent from outside hospitals. In addition, limited interaction among different specialists was frequently mentioned. The results of the questionnaire indicated a high level of agreement on several proposed actions, such as the evaluation of regional differences, the use of organisational models already adopted in some Italian oncology networks, the introduction of new biomarkers together with dedicated training activities, and the use of digital tools to support the discussion of complex cases. All survey items reached the predefined consensus threshold.

**Conclusions:**

This study identified several organizational and diagnostic challenges affecting the implementation of immunotherapy in AGC. Participants highlighted a number of potential priorities for improvement, including optimization of diagnostic workflows, stronger multidisciplinary collaboration, and greater coordination across centres. These findings may inform future initiatives aimed at reducing variability in clinical practice and supporting equitable access to innovative treatments.

**Supplementary Information:**

The online version contains supplementary material available at https://doi.org/10.1007/s12029-026-01557-0.

## Introduction

Gastric cancer remains a highly aggressive malignancy, representing the fourth leading cause of cancer death in the world and the fifth most common malignant cancer [1]. It’s characterized by a dismal prognosis due to both its propensity for recurrence following radical surgery and its frequent diagnosis at advanced stages [1]. In Europe, five-year survival rates are approximately 25%, placing gastric cancer among those with the poorest outcomes. In Italy, nearly 14,000 new cases of gastric cancer are diagnosed annually, with five-year survival rates of approximately 30% in men and 35% in women [2]. For early-stage gastric cancer, surgical resection remains the primary treatment modality. Despite this, clinical outcomes are often limited by a high risk of recurrence even after surgery and by the lack of effective options for patients who develop resistance to established therapies. Additionally, a significant proportion of patients present with advanced disease at diagnosis [3].

For patients with advanced disease, systemic chemotherapy still represents the primary treatment modality, offering a significant improvement in overall survival compared with best supportive care [2]. Recently, immune checkpoint inhibitors (ICIs) have emerged as new standard treatment in several malignancies, including advanced gastric cancer (AGC), showing favourable clinical benefit [4–6]. When combined with chemotherapy, ICIs have demonstrated both statistically significant and clinically meaningful improvements in overall survival [2–8]. In AGC the combination of immune checkpoint inhibitors with chemotherapy has shown particularly promising results. The PD-L1 combined positive score (CPS) has been increasingly developed as a possible predictive biomarker of response to immunotherapy and is being regularly used as a stratification marker in clinical trials [9–12]. This predictive score has been defined as the number of PD-L1-positive cells (tumour cells, macrophages, and lymphocytes), divided by the total number of tumour cells and multiplied by 100. The score varies between CPS ≥ 1, CPS ≥ 5, and CPS ≥ 10. In gastric cancer, PD-L1 CPS ≥ 1 generally defines PD-L1-positive tumours [9–13]. Recent studies have highlighted variability in PD-L1 assessment across different assays and observers, raising concerns regarding the consistency of immunotherapy eligibility assessment in routine clinical practice [14–17].

Despite the growing evidence supporting immunotherapy in advanced gastric cancer, less attention has been devoted to the organizational and diagnostic factors that may influence its implementation in routine clinical practice. In this context, we conducted a two-phase qualitative study involving oncologists and pathologists from five national referral centres. The study aimed to identify organizational and diagnostic barriers affecting immunotherapy implementation and to explore potential priorities for improving diagnostic workflows, multidisciplinary collaboration, and coordination across centres.

## Methods

This work was structured in two sequential phases. The first phase consisted of a semi-structured focus group aimed at exploring current clinical practice, diagnostic pathways, and organizational barriers related to immunotherapy implementation in advanced gastric cancer (AGC). The second phase consisted of a structured consensus survey based on the themes identified during the focus group and was used to assess which organizational and diagnostic priorities were considered most relevant by participating experts.

### Phase 1

Qualitative exploratory analysis: For the first phase, we set up a focus group with 13 specialists—oncologists and pathologists—from five Italian referral centres with established experience in the diagnosis and management of advanced gastric cancer. Centres were selected to ensure representation of different organizational settings and geographical areas, while including professionals directly involved in biomarker assessment and treatment decision-making.

The session was moderated by a clinical psychologist with experience in qualitative research and followed a semi-structured discussion guide. The guide explored four predefined thematic areas: access to biomarker testing (e.g., availability and timeliness of PD-L1 assessment including CPS evaluation and MSI testing, pre-analytical challenges); clinical experience with immunotherapy (e.g., perceptions of efficacy and safety, patient selection criteria, management of adverse events); organizational barriers and multidisciplinary workflows (e.g., integration of testing results into therapeutic decision-making and functioning of multidisciplinary teams); and strategies to optimize access and care delivery (e.g., proposals to improve local and regional pathways and identification of best practices).

The discussion lasted approximately two hours and was audio-recorded and transcribed. The transcript and moderator notes were reviewed using a thematic analysis approach. While the discussion guide provided an initial analytical framework, recurring concepts emerging from participants’ contributions were identified and grouped into broader thematic areas through iterative review of the discussion content. The preliminary thematic areas were subsequently reviewed and discussed within the study team and were used to inform the development of the consensus survey conducted during the second phase of the study.

### Phase 2: Consensus

Themes emerging from the qualitative phase were used to develop a structured online questionnaire aimed at assessing the level of agreement on the main organizational and diagnostic priorities identified during the focus group. Following the focus group, participants were invited to identify additional healthcare professionals with recognized expertise in the management of advanced gastric cancer. These experts were subsequently invited to participate in the survey to provide additional multidisciplinary perspectives on the issues identified during the focus group.

The questionnaire was organized into five thematic areas and included 18 items reflecting the domains discussed during the focus group: pre-analytical processes and testing quality (e.g., turnaround times, quality indicators for PD-L1 testing, inter-observer variability); multidisciplinary care pathways (e.g., early integration of biomarker testing, collaboration between clinicians and pathologists); regional variability and best practices (e.g., monitoring local disparities, sharing successful organizational models); emerging biomarkers (e.g., Claudin 18.2 (CLDN18.2), use of new predictive markers, regulatory uncertainties); and digital solutions and professional training (e.g., virtual slide platforms and continuing education initiatives).

The survey was distributed to 19 multidisciplinary experts, including oncologists, pathologists, pharmacists, surgeons, and endoscopists. Of the 19 invited experts, 9 completed the questionnaire.

Responses were collected using 5-point Likert scales to assess the level of agreement with the organizational barriers and improvement strategies identified during the qualitative phase. Descriptive statistics (frequencies and percentages) were used to summarize responses.

Agreement was defined as ratings of 4 or 5 on the Likert scale. Consistent with thresholds commonly adopted in consensus-based healthcare research, agreement ≥ 70% was considered indicative of substantial consensus.

### Ethical Considerations

No patients were involved in the study, and no individual patient-level clinical data or identifiable personal information were collected. Participants contributed in their professional capacity as clinical experts. Therefore, formal ethics committee approval was not required.

## Results

### Qualitative Findings

Five main themes emerged from the focus group discussion, reflecting organizational and diagnostic issues encountered during the implementation of immunotherapy in AGC: pre-analytical processes and diagnostic timelines, multidisciplinary team functioning, regional variability, emerging biomarkers, and digital tools and professional development.

1. Pre-analytical processes and diagnostic timelines: Delays in biomarker testing, particularly PD-L1 assessment, emerged as a recurrent concern during the discussion. Turnaround times varied across centres and were reported to be longer when samples were obtained from external hospitals or when biopsy material was limited or suboptimal. Several participants emphasized that pre-analytical factors, including tissue fixation, sample adequacy, and specimen transport, may influence the reliability and timing of biomarker assessment. The lack of shared performance indicators for pre-analytical processes was perceived as a source of variability between institutions.

“*Delays often depend more on organizational gaps than on technical issues*.”

2. Multidisciplinary team (MDT): MDTs were active in all participating centres and were considered essential for coordinating patient management and supporting multidisciplinary decision-making. Regional differences in access to diagnostic technologies and innovative therapies were frequently discussed. While overall satisfaction with MDT functioning was high, some participants noted occasional challenges in ensuring the continuous participation of all team members due to heavy workloads.

*“The patient needs to be discussed from the very start, already during the diagnostic phase. Otherwise, we risk missing key steps along the way.”*

3. Regional disparities and access inequities: participants highlighted notable regional differences in access to diagnostic technologies and innovative therapies, raising concerns about equity of care. This variability was perceived as a potential source of unequal access to biomarker testing and, consequently, to appropriate treatment pathways. Participants suggested that regional or national monitoring systems could help identify differences in diagnostic workflows and support greater harmonization across centres.

*“There are patients who have access only in certain regions”*

4. Emerging biomarkers and future challenges: while participants recognized the potential of emerging biomarkers (e.g., CLDN18.2), they also reported a lack of experience and structured training in their application. Barriers included limited experience, uncertainty regarding reimbursement and availability, and the need for specific training and shared clinical-pathological criteria. Several contributors noted that the introduction of new biomarkers would require dedicated training initiatives and shared multidisciplinary assessment criteria.

*“Training is needed to integrate these new biomarkers into routine clinical practice.”*

5. Digital tools and professional development: Participants highlighted the lack of structured spaces for discussion among specialists as a barrier to coordinated care. This gap was seen as particularly critical in the context of complex diagnostics and the integration of emerging biomarkers. Digital platforms were suggested as a potential solution. In addition, structured education programs were considered essential to support multidisciplinary teams navigating the evolving landscape of immunotherapy.

*“We need dedicated spaces for pathologists to discuss difficult cases and learn together.”*

### Consensus Survey Findings

To further assess the level of agreement on the priorities emerging from the focus group, a structured survey was sent to 19 experts from different specialties (oncologists, pathologists, pharmacists, surgeons, and endoscopists). Nine experts completed the survey (response rate: 47%), offering a broader multidisciplinary perspective.

Agreement levels were consistently high across the 18 survey items, ranging from 88.9% to 100%. All items reached the pre-defined consensus threshold of ≥ 70%. Full item-level results, including the wording of each question, thematic area, consensus percentage, and number of respondents assigning a score of 4 or 5, are reported in Supplementary Table S1.

The highest levels of agreement were observed for initiatives aimed at improving standardization and coordination across centres. These included recommendations on pre-analytical turnaround times, monitoring of external samples, structured peer support among pathologists, early MDT discussion, and closer collaboration between oncologists and pathologists in defining testing strategies. High agreement was also observed for actions addressing interregional variability and for the systematic assessment and implementation of CLDN18.2-related testing where appropriate.

Agreement levels of 88.9% were observed for the development of quality indicators for PD-L1 testing, joint assessment of PD-L1, MSI, and HER2 at diagnosis, consolidation of regional best practices, establishment of a national observatory on immunotherapy use, multidisciplinary evaluation of emerging biomarkers, and the use of digital platforms for virtual slide sharing.

Additional priorities from open-ended responses.

Open-ended survey responses provided further detail on potential actions to improve AGC management (box 1). Respondents emphasized the need to make HER2, MMR/MSI, and PD-L1 assessment a systematic component of pathology reporting across centres. Other priorities included dedicated training in predictive diagnostics, stronger collaboration between clinicians and pathologists, greater involvement of molecular biologists in diagnostic pathways, the development of reference centres for molecular testing, and more rapid access to centralized molecular information.

Box 1. Shared priorities for implementing immunotherapy in advanced gastric cancer.

**Table:** 

1.Standardize pre-analytical processes for biopsy collection and fixation
2.Develop tracking systems for pre-analytical phases, including external samples
3.Create structured peer discussion forums among pathologists
4.Strengthen multidisciplinary collaboration from the diagnostic stage
5.Leverage digital platforms and regional hub-and-spoke networks to improve access
6. Establish shared diagnostic-therapeutic pathways at national and regional levels

## Discussion

This study identified several organizational and diagnostic barriers affecting the implementation of immunotherapy in AGC, particularly within diagnostic workflows and multidisciplinary care pathways. These findings are consistent with challenges previously described in the literature, while providing additional insight into how such issues are perceived and managed within Italian referral centres. The perspectives collected from specialists directly involved in AGC management help contextualize the practical factors that may influence the delivery of immunotherapy across different healthcare settings.

A key issue raised by focus group participants was the variability and delays encountered in PD‑L1 testing. By contrast, HER2 and MSI assessments were generally processed more efficiently. Delays in PD‑L1 testing were often attributed to organizational challenges, including inconsistent quality and quantity of biopsy samples—especially when sourced from external facilities—non-standardized pre-analytical procedures, and logistical bottlenecks in specimen transport Participants noted that these factors may affect antigen preservation and, consequently, the reliability of test results. The absence of uniform criteria for monitoring pre-analytical processes was also perceived as a source of variability across institutions.

These issues in testing workflows reported by our panel are consistent with broader systemic challenges that have been extensively described in the literature. Mastracci et al. (2022) and Angerilli et al. (2023) [18, 19] noted inconsistent thresholds and scoring approaches—CPS versus TPS—along with major variation between observers. They also reported how results can differ from lab to lab because of the use of distinct platforms like 22C3, SP263, and SP142. Similar concerns have been reported by Yeong et al. (2022) who observed differences in CPS values according to the assay used, with the 28–8 assay tending to generate higher CPS scores than 22C3 [15]. Robert et al. (2023) added further weight to these concerns. In their international study of 12 pathologists reviewing 100 pretreatment biopsies, agreement levels were only moderate (ICC 0.45–0.57). For some CPS elements—PD‑L1‑positive immune cells and total viable tumor cells—the agreement was even weaker (ICC 0.19 and 0.09, respectively) [20].

Taken together, these observations highlight the importance of standardized protocols and quality-control measures to support the consistency and reliability of PD-L1 testing in routine clinical practice.

However, despite the availability of national and international guidelines recommending standardized PD‑L1 testing procedures [21–23], their implementation across healthcare systems remains inconsistent. Participants in our study reported that these recommendations are not fully adopted in practice due to organizational constraints, including limited training opportunities and a shortage of dedicated gastrointestinal pathologists.

Beyond technical hurdles, other elements—like intra-tumoral heterogeneity, regions of ulceration or chronic inflammation, and even the use of older tissue samples (over five years)—can make PD‑L1 assessment less reliable [18]. In response to these challenges, several participants suggested greater centralization of selected diagnostic activities. According to participants, such an approach could contribute to greater standardization of testing procedures and potentially reduce variability across centres.

The priorities identified by participants—including reducing turnaround times for biomarker testing, strengthening the integration of biomarker information within multidisciplinary discussions, promoting quality indicators, and supporting training and digital collaboration initiatives—reflect broader challenges associated with the implementation of precision oncology in routine clinical practice. The introduction of emerging biomarkers was also viewed as requiring appropriate organizational structures and professional training to support their integration into routine practice.

The priorities emerging from this study are aligned with international standards, such as the ESMO Clinical Practice Guidelines [23], which stress the importance of strong biomarker testing infrastructures, multidisciplinary collaboration, and standardized diagnostic workflows as key pillars of high-quality cancer care. On a regional level, initiatives like the Piemonte Oncology Network [24] and the Lombardy Oncology Network [25]—where a regional Molecular Tumor Board (MTB‑R) coordinates shared diagnostic‑therapeutic pathways and ensures uniform practices across authorized laboratories— offer concrete examples. In both cases, the Molecular Tumor Board brings together oncologists, pathologists, molecular biologists, bioinformaticians, and even patient advocates to set diagnostic protocols, track critical performance metrics—such as turnaround times and adherence to testing standards—and promote consistent practices across institutions.

The priorities identified by participants were largely aligned with current international recommendations and with organizational models already adopted in some regional oncology networks. This convergence suggests that the challenges discussed during the study may not be limited to the participating centres alone. Similar organizational and diagnostic issues have been described in a variety of healthcare settings, suggesting that these challenges remain relevant as access to biomarker-driven therapies continues to expand.

While the emerging challenges have been widely documented in the literature, our study captures the experience of clinicians directly involved in the management of patients with AGC within Italian referral centres. The findings provide insight into how these issues are experienced in daily clinical practice and identify areas that participating professionals considered particularly important for improving care pathways. Although our study was not designed to assess clinical outcomes directly, participants repeatedly emphasized that timely biomarker assessment represents a prerequisite for treatment planning in advanced gastric cancer. Delays in PD-L1 testing were perceived as particularly relevant because treatment decisions increasingly rely on the availability of biomarker information. In this context, inefficiencies in diagnostic workflows may delay multidisciplinary discussion and treatment planning, especially when samples originate from external institutions or require additional pathological review. Timely access to biomarker information was also considered important for ensuring that potentially eligible patients could be evaluated for appropriate treatment options without avoidable delays. While the direct impact of these factors on clinical outcomes was beyond the scope of the present study, participants consistently identified them as practical barriers to the timely delivery of biomarker-driven care.

Beyond their organizational implications, these aspects may also influence the overall patient experience, an area that is receiving increasing attention in oncology and healthcare decision-making alongside traditional measures of clinical benefit [26].

From a healthcare planning perspective, the findings highlight the importance of organizational factors in supporting the implementation of immunotherapy. Efficient diagnostic pathways, timely biomarker assessment, and effective multidisciplinary collaboration may facilitate the appropriate use of innovative therapies in routine clinical practice. In this context, the organizational issues identified by participants may be relevant not only for clinical practice, but also for healthcare policy and health technology assessment (HTA). Increasing attention is being paid to implementation processes, care pathways, and patient-centred outcomes as complementary elements to traditional measures of clinical effectiveness.

## Limitations

The findings of this study should be interpreted in light of several limitations. The qualitative phase involved specialists from five Italian referral centres with established expertise in AGC management. Although these centres were selected to capture different organizational settings and geographical areas, their experiences may not fully represent practices across all healthcare contexts. Similarly, the survey was completed by 9 of the 19 invited experts. While this response rate is comparable to that reported in many professional online surveys [27], the possibility of non-response bias cannot be excluded, and the high levels of agreement observed should be interpreted with appropriate caution.

Another important consideration is that the study explored the perceptions and experiences of healthcare professionals rather than objective measures of implementation performance or patient-level outcomes. As a result, the findings provide insight into how organizational and diagnostic challenges are perceived in routine practice, but they do not allow conclusions regarding the direct impact of these factors on treatment outcomes. Finally, given the exploratory nature of the study and the purposive selection of participants, the findings should be viewed primarily as hypothesis-generating. Further research involving a broader range of centres and real-world organizational data would help to validate and extend these observations.

## Conclusions

In this two-phase analysis, we identified several barriers and practical priorities for improving how immunotherapy is delivered for AGC in the Italian healthcare system. The findings highlight a number of priority areas, including timely biomarker assessment, multidisciplinary integration, quality monitoring, professional training, and the use of digital tools to support collaboration across centres. While exploratory in nature, the study provides a structured overview of issues that clinicians consider important for optimizing the delivery of immunotherapy in routine practice. These findings may support future organizational initiatives, policy discussions, and research focused on strengthening biomarker-driven care pathways and reducing variability in their implementation.

## Supplementary Information

Below is the link to the electronic supplementary material.

Supplementary file1 (DOCX 14 KB)

## Data Availability

The datasets generated and/or analysed during the current study consist of anonymized expert discussions and survey responses and are available from the corresponding author on reasonable request.
