# Supplementary material for: Barriers and Priorities for Immunotherapy Implementation in Advanced Gastric Cancer: Findings from a Two-Phase Qualitative Study
Source: J Gastrointest Cancer. 2026 Sep 3;57(1):208. doi: 10.1007/s12029-026-01557-0 (PMC13541941; doi:10.1007/s12029-026-01557-0)
Supplement: Supplementary file 1 [file 12029_2026_1557_MOESM1_ESM.docx]

Supplementary Materials

## **Table S1 – Detailed questions with scores and consensus percentages**

| **Question** | **Macroarea** | **Consensus percentage** | **Score (4-5 ratings out of 9)** |
| --- | --- | --- | --- |
| 1.Promote the systematic adoption of guidelines on pre-analytical turnaround times | Pre-analytical | 100.0% | 9/9 |
| 2. Monitor pre-analytical turnaround times also for external samples | Pre-analytical | 100.0% | 9/9 |
| 3. Develop quality indicators for PD-L1 testing | Pre-analytical | 88.9% | 8/9 |
| 4. Implement structured peer support among pathologists for PD-L1 scoring | Pre-analytical | 100.0% | 9/9 |
| 5. Include multidisciplinary team (MDT) discussion already at the stage of endoscopic diagnosis | Multidisciplinary | 100.0% | 9/9 |
| 6. Foster active collaboration between oncologists and pathologists to define testing strategies | Multidisciplinary | 100.0% | 9/9 |
| 7. Perform PD-L1, MSI, and HER2 testing jointly at the initial diagnostic stage | Multidisciplinary | 88.9% | 8/9 |
| 8. Develop structured training programs for pathologists on PD-L1 testing | Digital tools and professionl training | 100.0% | 9/9 |
| 9. Involve clinicians in joint updates and training sessions with pathologists | Digital tools and professionl training | 100.0% | 9/9 |
| 10. Monitor interregional variability through national audits | Regional variability | 100.0% | 9/9 |
| 11. Systematize and scale up consolidated regional best practices | Regional variability | 88.9% | 8/9 |
| 12. Establish a permanent observatory on the use of immunotherapy | Emerging biomarkers | 88.9% | 8/9 |
| 13. Systematically assess CLDN18.2 even in PD-L1-negative patients | Emerging biomarkers | 100.0% | 9/9 |
| 14. Adopt more rational criteria for selecting patients for immunotherapy | Emerging biomarkers | 100.0% | 9/9 |
| 15. Evaluate emerging biomarkers through joint clinical-pathological assessment | Emerging biomarkers | 88.9% | 8/9 |
| 16. Prioritize organizational efforts on pre-analytical phase and biomarker scoring | Emerging biomarkers | 100.0% | 9/9 |
| 17. Promote digital platforms for virtual slide sharing | Digital tools and professionl trainig | 88.9% | 8/9 |
| 18. Address CLDN18.2 challenges due to regulatory uncertainty | Emerging biomarkers | 100.0% | 9/9 |
